# Natural Products as Modulators of the Proteostasis Machinery: Implications in Neurodegenerative Diseases

**DOI:** 10.3390/ijms20194666

**Published:** 2019-09-20

**Authors:** Karina Cuanalo-Contreras, Ines Moreno-Gonzalez

**Affiliations:** 1 The Mitchell Center for Alzheimer′s Disease and Related Brain Disorders, Department of Neurology, The University of Texas Houston Health Science Center at Houston, Houston, TX 77030, USA; 2 Departamento Biologia Celular, Genetica y Fisiologia, Instituto de Investigacion Biomedica de Malaga-IBIMA, Facultad de Ciencias, Universidad de Malaga, 28031 Madrid, Spain; 3 Networking Research Center on Neurodegenerative Diseases (CIBERNED), 28031 Madrid, Spain

**Keywords:** proteostasis, neurodegeneration, chaperones, autophagy, ubiquitin-proteasome, unfolded protein response, natural compounds

## Abstract

Proteins play crucial and diverse roles within the cell. To exert their biological function they must fold to acquire an appropriate three-dimensional conformation. Once their function is fulfilled, they need to be properly degraded to hamper any possible damage. Protein homeostasis or proteostasis comprises a complex interconnected network that regulates different steps of the protein quality control, from synthesis and folding, to degradation. Due to the primary role of proteins in cellular function, the integrity of this network is critical to assure functionality and health across lifespan. Proteostasis failure has been reported in the context of aging and neurodegeneration, such as Alzheimer’s and Parkinson’s disease. Therefore, targeting the proteostasis elements emerges as a promising neuroprotective therapeutic approach to prevent or ameliorate the progression of these disorders. A variety of natural products are known to be neuroprotective by protein homeostasis interaction. In this review, we will focus on the current knowledge regarding the use of natural products as modulators of different components of the proteostasis machinery within the framework of age-associated neurodegenerative diseases.

## 1. Proteostasis Failure in Aging and Neurodegenerative Diseases

The proteostasis network is composed of a series of interconnected elements that assure correct protein functionality and degradation [1]. It starts when polypeptide chains are synthetized in the ribosome and fold with the help of chaperones and co-chaperones. Newly folded proteins are transported to their appropriate locations and once their life cycle finishes, they are degraded either by the ubiquitin proteasome system (UPS) or the autophagy machinery. Proteostasis network imbalance plays a key -if not causative- role in many age-related pathologies [2]. Age is the most relevant risk factor for neurodegenerative diseases including Alzheimer’s disease (AD), Parkinson disease (PD), frontotemporal dementia (FTD) and several other forms of proteinopathies [3]. Although there is no consensus in the field regarding the molecular mechanisms that explain their augmented incidence in the elderly brain, a common feature of all these diseases is the accumulation of abnormal protein aggregates in the form of oligomers and inclusions, suggesting that general mechanisms controlling proteostasis may underlay the etiology of these diseases [4]. Recent hypotheses suggest that a progressive reduction in the repair capacity of the proteostasis network may generate a “pathological aging” that results in protein aggregation and higher incidence of neurodegenerative disease [5,6,7,8]. Cerebral aging involves a range of cellular and molecular alterations related to proteostasis impairment such as increased oxidative stress [9], altered autophagy machinery [10], accumulation of ubiquitinated protein aggregates [11], and impaired signaling by numerous neurotransmitters and neurotrophic factors [12]. The endoplasmic reticulum (ER) is an essential compartment of the proteostasis network, which is also disturbed by the aging process [4]. Importantly, functional studies indicate that altered proteostasis at the level of the ER is one of the major contributors to aging [4,13]. Several harmful stimuli, such oxidative stress and disturbances in the secretory pathway may lead to accumulation of unfolded or misfolded proteins at the ER lumen, thus activating the ER stress response [14].

The most prominent pathological hallmarks of AD are the extracellular accumulation of amyloid β (Aβ) peptides in the form of plaques and the intracellular accumulation of hyper-phosphorylated tau (ptau) proteins as neurofibrillary tangles (NFTs) [15], whereas in PD, α-synuclein tends to misfold and accumulate inside dopaminergic neurons, leading to Lewy bodies formation [16]. Formation of misfolded proteins as oligomers, proto-fibrils and fibrils leads to the accumulation of amyloid deposition and spreading to affected areas [17,18]. Several intrinsic and extrinsic factors that alter proteostasis cause a decreased protein quality control, contributing to the accumulation of damaged proteins. If not rescued, this condition can lead to protein misfolding disorders, such as AD and PD [19,20,21,22,23]. For instance, a growing amount of evidence indicate that the activity of the molecular chaperones -Hsp60, Hsp70 and Hsp90- is compromised in age-related neurodegenerative diseases [24,25]. The fact that the expression of Hsp60 and Hsp70 is decreased in AD animal models [26], suggests that impairments in the folding pathways play a key role in promoting age-related neurodegeneration. In prion diseases, reduction of the molecular chaperone GRP78/BiP expression leads to the acceleration of the pathology [27]. Alterations in the major protein degradation pathways have a major involvement as well. For instance, the reduction in the activity of the UPS through the manipulation of various UPS components (Rpt2, Rpt3, ubiquitin) causes deposition of pathological misfolded proteins and subsequent neurodegeneration in experimental models, resembling what is observed in AD and PD [28,29,30]. In addition, neurodegenerative diseases have in common autophagic failure [31,32]. The inhibition of the autophagy response is known to exacerbate protein toxicity and accelerate disease progression [33,34,35]. The genetic and pharmacological activation of the autophagy has shown to improve the clearance of AD and PD misfolded aggregated proteins [36,37,38]. Therefore, one can conclude that boosting up the elements of the proteostasis machinery is a promising broad-spectrum therapeutic approach, with the potential to treat or revert not only age-associated neurodegeneration, but a variety of protein misfolding disorders.

## 2. Chaperone System

Chaperones are highly conserved proteins that assist and mediate the achievement of the proper three-dimensional conformation of proteins. They bind and stabilize unfolded polypeptides, aiding their folding during synthesis and inter-organelle transport [39]. Chaperones play important roles during stress response, hence they are known as heat shock proteins (Hsp). Hsp are classified by their molecular mass (Hsp32, Hsp27, Hsp40, Hsp60, Hsp70, Hsp90). They possess a substrate binding domain that transiently binds to hydrophobic regions of polypeptides, shielding them from undesired intermolecular interactions that could interfere with their adequate folding [40]. The capacity of the Hsp is overloaded during chronic cellular stress, proteotoxic conditions and disease. For instance, Hsp failure has been observed in the context of neurodegenerative disorders, such as AD, PD and Huntington’s disease [41]. Notably, the solely over-expression of different Hsp members has been able to rescue in vivo neuronal toxicity in different models [42,43,44,45]. With this in mind, the pharmacological activation of Hsp represents an interesting therapeutic approach to treat neurodegeneration.

To date, several natural products have been identified as Hsp modulators. Among them, the potent phytochemical curcumin, a polyphenol of the plant *Curcumin longa*, has shown the ability to induce the in vitro (rat glioma cells, rat liver cells, and mouse fibroblasts) and in vivo (heat-stressed rats) expression of Hsp27 and Hsp70 under proteotoxic conditions, through the formation of an intermediate form of Hsf1 (heat shock factor 1) [46,47]. Although the administration of curcumin in animal models of neurodegenerative diseases has proven to be beneficial [48,49] and has no major side effects in humans, some clinical trials show no evidence of efficacy in ameliorating memory impairment nor reducing levels of amyloid in blood, suggesting a low bioavailability of curcumin following oral administration [50,51]. Several other nutraceuticals have the ability to boost the chaperone system, such as the proanthocyanidins present in cranberry extract. When administered to an AD nematode model, they delayed Aβ toxicity through the activation of Hsf1, which is a master regulator of Hsp expression [52]. Another interesting phytochemical is celastrol (extracted from the thunder god vine, *Tripterygium wilfordii*). Celastrol administration to aged mature cortical cultures induced the expression of Hsp70, Hsp32 and Hsp27 [53]. To highlight the in vivo neuroprotective activity of this natural product, intraperitoneal as well as subcutaneous administration in AD mice reduced Aβ pathology [54]. No clinical trials have been performed using cranberry extract or celastrol to treat AD.

Paeoniflorin is an herbal compound isolated from the perennial flowering plant *Paeonia lactiflora* and the fern *Salvinia molesta*. This phytochemical bears the ability to induce Hsp expression through activation of Hsf1 and promotes thermotolerance in mammalian cell culture as well [55]. Another major constituent of the same herbal medicines is Glycyrrhizin, which can be found in the liquorice root. Several properties have been attributed to Glycyrrhizin, such as antiviral, anti-inflammatory, and anti-allergic. In fact, it has been tested in over 20 different clinical trials related with liver diseases with positive outcomes, but none of them evaluated its effect in neurodegenerative diseases. In the case of the heat shock response, Glycyrrhizin is not able to promote the expression of Hsp itself, however it enhances their induction, making it an interesting compound that could potentially be used in combination with activators of the heat shock response [55]. Some natural occurring antibiotics have Hsp induction properties too. Geldanamycin is a 1,4-benzoquinone ansamycin natural antibiotic compound isolated from the bacterial species *Streptomyces hygroscopicus*. When administered to mammalian cells expressing huntingtin exon 1 protein, it induces the expression of Hsp40, Hsp70 and Hsp90. The consequent activation of the heat shock response causes a marked inhibition on huntingtin aggregation [56]. In patients with primary brain tumor or brain metastases, geldanamycin induces Hsp70 with minimal toxicity [57]. Therefore, this compound bears the potential to treat disease-associated protein aggregation. Another antibiotic compound isolated from *Streptomyces* is herbimycin-A. Herbimicyn-A has the ability to induce the expression of Hsp72 thereby protecting cell cultures from heat stress [58]. Radicicol is a natural macrocyclic compound biosynthesized and isolated from the nematophagous fungi *Pochonia chlamydosporia*. This compound protects primary cell cultures against stressful conditions, by inducing the heat shock response in a HSF-1 related manner, following a similar mechanism than Herbimicyn and Geldanamycin [59]. In addition to this natural antibiotics, several other compounds have shown the ability to boost the chaperone system. While there are not reports yet on their action in the context of neurodegeneration or even clinical trials, they represent promising candidates to restore proteostasis balance and may have potential to delay the onset or treat diseases such AD or PD. One example is withaferin, a lactone derived from the plant *Vassobia breviflora*. Withaferin enhanced the heat shock response through Hsp70, Hsp32 and Hsp27 upregulation in a cancer model [60] and it is reported to ameliorate symptoms in schizophrenia patients with minimal side effects [61]. Shikonin is another potential candidate to treat proteinopaties. Its ability to induce Hsp70 in a human lymphoma cell model was discovered through a screening of chemical inducers derived from medicinal plants. Shikonin is present in the roots of *Lithospermum erythrorhizon* and it bears antibacterial, anti-inflammatory and anticancer activities as well [62]. Edible gastropods seem to be an interesting source of compounds with potential to modulate proteostasis response. As an example, the derivative 6-bromoindirubin-3-oxime, an indirubin present in mollusks, increased proteasome subunits and Hsp70 expression, with a consequent increase in healthspan and lifespan in *Drosophila* [63]. Few clinical trials have tested the efficacy and safety of indirubins, such as indigo naturalis extract. Although it can be considered a safe therapy [64], these studies have been tested in psoriasis patients and therefore, its bioavailability remains unknown.

## 3. Autophagy

Autophagy is a highly conserved homeostatic clearance mechanism. Is in charge of the degradation of damaged proteins, cytosolic components and organelles. It involves the lysosomal system and contributes to the regulation of metabolism, healthspan and longevity. Cellular autophagy activity is present at basal levels, however is particularly stimulated under stress conditions, as a protective mechanism to assure survival and homeostasis [65,66]. Autophagy impairment has been reported in several pathologies, from neurodegeneration to cancer [67,68]. Autophagy targets the degradation of misfolded aggregated proteins considered hallmarks of different proteinopathies [67,69]. However, during disease, the autophagy machinery fails, with deleterious cellular consequences [31]. Several studies have pinpointed a downregulation of important components of the autophagy pathway during AD and PD, such as Beclin 1 [70], as well as alteration in vesicle trafficking and inhibition of autophagic vesicles [71]. Notably, the genetic and pharmacological induction of autophagy has the ability to reduce the accumulation of misfolded proteins and has been associated to amelioration of these disorders [36,37,72]. In this regard, polyphenolic compounds are known potent activators of the autophagy response. As an illustration, the red wine polyphenol quercetin prevents Aβ associated aggregation and its obnoxious consequences through modulation of autophagy, both in nematodes [73] and murine models of AD [74] and PD [75]. Currently, there is a clinical trial to determine the brain penetration of quercetin to potentially treat AD patients using a senolytic therapy. Kaempferol is another potent polyphenol found in different dietary sources such as grapes and tomatoes. In vitro kaempferol treatment increases LC3-II, an autophagosome-bound microtubule-associated protein, and preserved the stratial glutamatergic response in a rat model of PD, positioning this natural product as an important enhancer of autophagy with promising therapeutic applications [76]. Interestingly, caffeine elevates LC3-II levels as well and has proven protective actions against AD and PD [77,78]. In fact, some studies suggest that drinking coffee may be associated with a decreased risk to develop AD and PD [79,80,81], however, no evidence has been obtained from randomized controlled trials about the beneficial effect of caffeine in neurodegenerative diseases to our knowledge. Resveratrol is another compound of interest present in grapes and berries. The fact that it can cross the blood-brain-barrier makes it an interesting candidate to treat neurodegeneration [82]. Among the many reported activities of resveratrol, it activates autophagy by up-regulating Sirtuin 1, a potent inductor of autophagy [83,84]. Moreover, in a clinical trial performed in AD patients, resveratrol modulates Aβ deposition and reduces inflammatory markers with no side effects [85,86]. In addition to this dietary sources, there is a growing amount of evidence demonstrating the beneficial effects of Mediterranean diet on age-associated neurodegeneration [87]. Olive oil is a significant component of this dietary regimen. Olive oil is enriched with the polyphenol oleuropein aglycone. The administration of oleuropein aglycone improved cognition and reduced amyloid deposition in a transgenic AD mouse model, mainly through activation of the autophagy [88]. A multitude of studies have study the effect of olive oil in combination with Mediterranean diet in an effort to evaluate its effect in patients with cognitive decline and dementia [89], including AD and PD, but none of them analyzed the capability of oleuropein aglycone to cross the blood-brain barrier (BBB), tolerance, biodistribution or its effect in treating neurodegenerative disorders. Another dietary molecule, present in high quantities on mushrooms and aged cheese, is spermidine. This compound induces autophagy and delays aging, the main risk factor for AD and PD, in humans and mice [90,91]. Glycoconjugate metabolites isolated from traditional medicine remedies are an interesting group of phytochemical compounds with properties to activate autophagy. For example, the ginseng derived steroid glycoside Rg2 is a potent inducer of in vitro and in vivo autophagy in an AMPK-ULK1 dependent [92]. In the same line, a derivative chemical compound from the root ginseng, 1-(3,4-dimethoxyphenethyl)-3-(3-dehydroxyl-20(s)-protopanaxadiol-3β-yl)-urea (DDPU), improved cognition and promoted neuroprotection in the APP/PS1 mouse model of AD. No clinical trials have been reported. DDPU targets different branches of the proteostasis network, as it has activity on both the ER stress and autophagy [93]. Berberine is a natural alkaloid isolated from *Rhizoma coptidis*, a traditional Chinese herbal medicine, with high distribution when administered orally, including the CNS, in pre-clinical studies [94]. When berberine was orally administered to a triple-transgenic AD mouse model, it promoted Aβ clearance through autophagy by increasing the levels of LC3-II. Phenotipically, berberine treatment significantly improved spatial learning and memory retention in the treated animals [95]. Corynoxine B joins the list of natural alkaloid molecules with autophagy-inducer properties in cellular and mouse AD models. Corynoxine B is an oxindole alkaloid present in the medicinal plant *Uncaria rhynchophylla,* a widely used Chinese traditional remedy. This compound was tested in cells expressing the APP_Swe_ mutation and intraperitoneally administered once a day to Tg2576 mice at 8 months of age. Corynoxine B treatment reduced Aβ levels by increasing LC3-II, lysosomal activation and changes in APP [96]. Surprisingly, the source of compounds with potential anti-neurodegenerative capacity is not limited to the ground. The study of marine organisms has helped to identify several compounds with the ability to modulate proteostasis. Among them, chromomycin A2, psammaplin A, and ilimaquinone induced the expression of autophagy, in the context of cancer [97]. It would be extremely interesting to test their effect on neurodegeneration, both in vitro and *in vivo*, as it will expand the sources of therapeutic molecules. As stated, autophagy is a major player in the cellular response to stress and turnover of damaged proteins. In view of its potential, targeting autophagy through the use of natural products is an emerging and promising field that requires further exploration.

## 4. Ubiquitin Proteasome System

The ubiquitin proteasome system (UPS) is the main responsible for degrading intracellular damaged proteins. Briefly, a subset of enzymes is involved in ubiquitin-tag the proteins that need to be degraded, this tag is then recognized by the proteasome -a multi-subunit barrel complex- for its proteolytic degradation [98]. Several natural compounds have been widely explored for their ability to decrease the activity of the UPS, especially in the context of cancer research [99]. However, relatively few have been studied for their capacity to activate the UPS. The mechanisms of action among them vary, for example, the natural compounds olein, linoleic acid, linolenic acid, ceramides, and oleuropein increase proteasome activity by exerting conformational changes that promote the entry of the substrate into the proteolytic chamber [100]. A derivative of linoleic acid has been reported to cross the BBB, tolerable, and safe, but specific studies to determine its potential to treat dementia are still needed [101]. On the other hand, dietary intake of linolenic acid seems to have no effect in other brain disorders such as stroke [102]. It remains to be determined whether this is due to a low brain penetrance of the compound in the CNS or lack of therapeutic potential in this specific disorder. Other natural molecules activate the proteasome by enhancing its catalytic activities, such as the lipid fraction of the algae *Phaeodactylum tricornutum* and the triterpene betulinic acid [103,104,105]. Two clinical trials are currently ongoing to determine the safety, tolerability and effectiveness of betulinic acid. The compounds present in the Chinese traditional herb *Corydalis bungeana* boost in vivo proteasomal activity by upregulation of the regulatory subunits [106]. In the same line, the polyphenol resveratrol enhances proteasome activity through increase on the expression of proteasome subunits and proteolysis in the brain of AD transgenic mice, protecting them against memory loss and enhancing cognition [107]. Quercetin is another polyphenolic compound that exhibits in vivo enhancing proteasome activity [108] and reduces Aβ-induced toxicity in a dose-dependent manner when administered to a *Caenorhabditis elegans* AD model [73]. Since impaired UPS activity is one of the main features present in all protein misfolding disorders, it will be interesting to explore the natural chemical space in the lookout for more activators.

## 5. Unfolded Protein Response

Three branches of a conserved signaling pathway collectively termed as the unfolded protein response (UPR) are triggered in response to the ER stress: ATF6 (activating transcription factor 6), PERK (PKR-like kinase), and IRE1 (inositol-requiring enzyme 1) [14]. UPR activation results in global protein synthesis reduction [109] and upregulation of genes involved in protein folding [14], which facilitates proper protein folding, therefore arresting protein aggregation. In the brain of AD, PD and FTD patients, levels of UPR markers are elevated [110,111]. This could represent an emergency response triggered by the ability of misfolded proteins to induce neuronal ER stress and activate the UPR [112]. However, when the ER response is chronically activated, proteostasis cannot be restored with devastating consequences for the brain, leading to synaptic impairment and neurodegeneration. In this line of thoughts, recent studies indicate that reduction of ER stress with chemical chaperones alleviate synapse and memory loss in experimental models of AD [111,112]. Levels of eIF2α phosphorylation are elevated in AD brains. PERK regulation decreases eIF2α phosphorylation levels and ameliorates memory impairment in AD and prion-infected mice [113,114]. On the other hand, activation of PERK increases tau phosphorylation [115], as well as ptau activates UPR [116]. IRE1 leads to the expression of XBP1 (X-box binding protein 1) that upregulates the expression of chaperones, increasing the size of the ER and promoting the degradation of misfolded proteins through the proteasome system [14,117]. It has been recently described that IRE1 signaling promotes AD progression whereas its deletion ameliorates learning and memory impairment as well as reduces amyloid deposition [118]. Furthermore, tau and Aβ accumulation has been also found associated with UPR activation by inhibition of ATF6 and ER-associated degradation (ERAD), likely through soluble oligomeric forms [116,119,120].

Few natural occurring compounds have been explored in regards to their ability to modulate the UPR in the context of neurodegeneration. Among them, Bajijiasu, a dimeric fructose isolated from the Chinese medicinal herb *Morinda officinalis,* has shown to exert protection against Aβ induced neurotoxicity by attenuation of ER stress in the hippocampus and cortex of APP/PS1 mice [121]. No clinical trials have been reported evaluating this compound. Kaempferol is phytoestrogen and one of the main components of *Ginkgo biloba* extract with the ability to inhibit ER stress and protect cells against apoptosis by upregulation of CHOP mRNA levels in vitro [122]. Clinical trial using *G. biloba* extracts indicate its symptomatic beneficial effects in patients with MCI, AD, and related dementia [123,124,125]. Honokiol is a promising biphenolic lignan isolated from the Magnolia tree that can cross the blood brain barrier and therefore represents an interesting candidate to treat neurodegeneration due to its high bioavailability. This lignan modulated ER-stress in the brain of mice, and reduced the levels of proinflammatory cytokines as well [126]. Its tolerance, safeness, biodistribution, and effectiveness has not been tested yet to treat brain disorders. More research is needed to evaluate the effect on neurodegeneration of other known modulators of ER-stress, as the current literature is quite limited. A special focus should be made on compounds with the ability to cross the blood brain barrier that can effectively target the cells that are compromised in this diseases.

## 6. Conclusions

**Figure 1:**
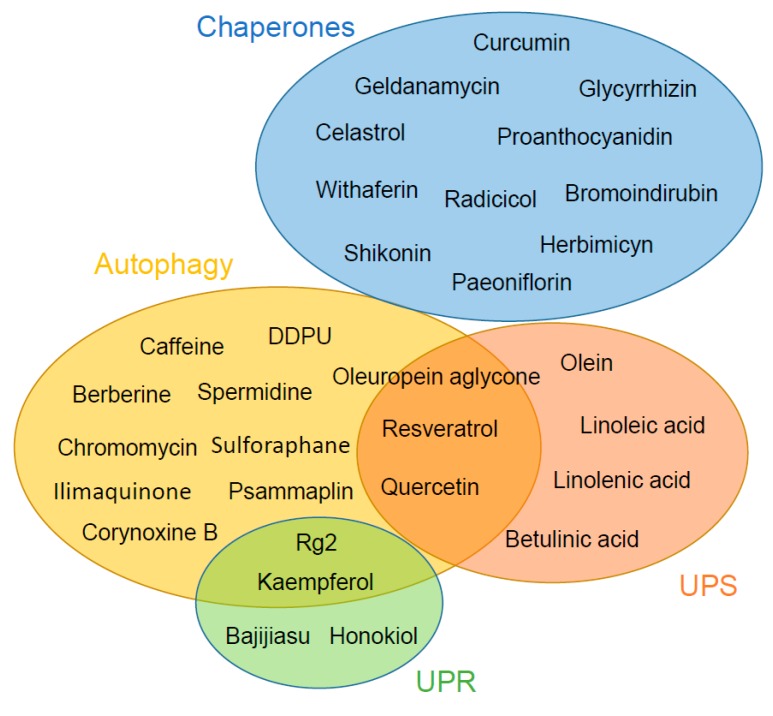
Schematic representation of natural compounds that positively regulate different elements of the proteostasis machinery. There is an extensive heterogeneity of chemical classes that compose the proteostasis-enhancing compounds, however we observed an enrichment in polyphenolic molecules. It is noted that oleuropein aglycone, resveratrol, and quercetin target the autophagy and the UPS, suggesting that they could be used as strong activators to restore the proteostasis network during aging and disease, whereas chaperones’ modifiers seem to exclusively interfere with this pathway.

Aging is the main risk factor for a variety of neurodegenerative disorders, such as AD and PD. Recent studies indicate that there is a dramatic age-associated collapse of proteostasis responses, leaving the cells vulnerable to physiological and environmental stressors, and more susceptible to disease. In the case of diseases associated with protein misfolding, the proteostasis machinery takes initial care of the aberrant protein aggregates. However, as the clearance ability gets compromised, the accumulated aggregates cause cellular toxicity, tissue dysfunction, and disease. Therefore, boosting up the proteostasis machinery by the use of natural compounds emerges as a potent pharmacological tool with promising effects to treat and protect against neurodegenerative disorders. In this study we compile a list of natural modulators of the proteostasis network (Figure 1). Not surprisingly, majority of them are of plant-origin. However it is remarkable to note that we report some compounds of marine-animal-origin as well. It is indeed necessary to explore more alternative sources of natural compounds. In addition, further studies are required to understand the precise mechanism of action of the natural proteostasis activators, their off-target effects and their in vivo bioavailability. We foresee that the development of innovative, natural and safe therapeutic strategies to tackle the accumulation of misfolded protein aggregates through the modulation of the proteostasis machinery, will have exceptional effects to prevent and treat disorders related to age-dependent protein aggregation.
